# Event‐based modeling in temporal lobe epilepsy demonstrates progressive atrophy from cross‐sectional data

**DOI:** 10.1111/epi.17316

**Published:** 2022-06-25

**Authors:** Seymour M. Lopez, Leon M. Aksman, Neil P. Oxtoby, Sjoerd B. Vos, Jun Rao, Erik Kaestner, Saud Alhusaini, Marina Alvim, Benjamin Bender, Andrea Bernasconi, Neda Bernasconi, Boris Bernhardt, Leonardo Bonilha, Lorenzo Caciagli, Benoit Caldairou, Maria Eugenia Caligiuri, Angels Calvet, Fernando Cendes, Luis Concha, Estefania Conde‐Blanco, Esmaeil Davoodi‐Bojd, Christophe de Bézenac, Norman Delanty, Patricia M. Desmond, Orrin Devinsky, Martin Domin, John S. Duncan, Niels K. Focke, Sonya Foley, Francesco Fortunato, Marian Galovic, Antonio Gambardella, Ezequiel Gleichgerrcht, Renzo Guerrini, Khalid Hamandi, Victoria Ives‐Deliperi, Graeme D. Jackson, Neda Jahanshad, Simon S. Keller, Peter Kochunov, Raviteja Kotikalapudi, Barbara A. K. Kreilkamp, Angelo Labate, Sara Larivière, Matteo Lenge, Elaine Lui, Charles Malpas, Pascal Martin, Mario Mascalchi, Sarah E. Medland, Stefano Meletti, Marcia E. Morita‐Sherman, Thomas W. Owen, Mark Richardson, Antonella Riva, Theodor Rüber, Ben Sinclair, Hamid Soltanian‐Zadeh, Dan J. Stein, Pasquale Striano, Peter N. Taylor, Sophia I. Thomopoulos, Paul M. Thompson, Manuela Tondelli, Anna Elisabetta Vaudano, Lucy Vivash, Yujiang Wang, Bernd Weber, Christopher D. Whelan, Roland Wiest, Gavin P. Winston, Clarissa Lin Yasuda, Carrie R. McDonald, Daniel C. Alexander, Sanjay M. Sisodiya, Andre Altmann, for the ENIGMA‐Epilepsy Working Group, Núria Bargalló, Emanuele Bartolini, Terence J. O’Brien, Rhys H. Thomas

**Affiliations:** ^1^ Centre for Medical Image Computing, Department of Medical Physics and Biomedical Engineering University College London London UK; ^2^ Stevens Neuroimaging and Informatics Institute, Keck School of Medicine University of Southern California Los Angeles California USA; ^3^ Centre for Medical Image Computing, Department of Computer Science University College London London UK; ^4^ Neuroradiological Academic Unit, UCL Queen Square Institute of Neurology University College London London UK; ^5^ Department of Psychiatry University of California, San Diego La Jolla California USA; ^6^ Department of Neurology Alpert Medical School of Brown University Providence Rhode Island USA; ^7^ Department of Molecular and Cellular Therapeutics Royal College of Surgeons in Ireland Dublin Ireland; ^8^ Department of Neurology and Neuroimaging Laboratory University of Campinas Campinas Brazil; ^9^ Department of Radiology, Diagnostic and Interventional Neuroradiology University Hospital Tübingen Tübingen Germany; ^10^ Neuroimaging of Epilepsy Laboratory Montreal Neurological Institute, McGill University Montreal Quebec Canada; ^11^ Multimodal Imaging and Connectome Analysis Laboratory, McConnell Brain Imaging Centre, Montreal Neurological Institute and Hospital McGill University Montreal Quebec Canada; ^12^ Department of Neurology Emory University Atlanta USA; ^13^ Department of Clinical and Experimental Epilepsy UCL Queen Square Institute of Neurology, University College London London UK; ^14^ Neuroscience Research Center, Department of Medical and Surgical Sciences Magna Græcia University of Catanzaro Catanzaro Italy; ^15^ Magnetic Resonance Image Core Facility August Pi i Sunyer Biomedical Research Institute, University of Barcelona Barcelona Spain; ^16^ Institute of Neurobiology National Autonomous University of Mexico Querétaro Mexico; ^17^ Epilepsy Program, Neurology Department Hospital Clinic of Barcelona Barcelona Spain; ^18^ August Pi i Sunyer Biomedical Research Institute Barcelona Spain; ^19^ Radiology and Research Administration Henry Ford Health System Detroit Michigan USA; ^20^ Department of Pharmacology and Therapeutics Institute of Systems, Molecular and Integrative Biology, University of Liverpool Liverpool UK; ^21^ FutureNeuro SFI Research Centre for Rare and Chronic Neurological Diseases Dublin Ireland; ^22^ Department of Radiology, Royal Melbourne Hospital University of Melbourne Melbourne Victoria Australia; ^23^ New York University Grossman School of Medicine New York New York USA; ^24^ Functional Imaging Unit, Department of Diagnostic Radiology and Neuroradiology Greifswald University Medicine Greifswald Germany; ^25^ Chalfont Centre for Epilepsy Chalfont St Peter UK; ^26^ Department of Neurology University Medical Center Göttingen Germany; ^27^ Cardiff University Brain Research Imaging Centre, School of Psychology Cardiff University Cardiff UK; ^28^ Institute of Neurology, Department of Medical and Surgical Sciences Magna Græcia University of Catanzaro Catanzaro Italy; ^29^ Department of Neurology University Hospital Zurich Zurich Switzerland; ^30^ Department of Neurology Medical University of South Carolina Charleston South Carolina USA; ^31^ Neuroscience Department University of Florence Florence Italy; ^32^ Wales Epilepsy Unit, Department of Neurology University Hospital of Wales Cardiff UK; ^33^ Neuroscience Institute University of Cape Town Cape Town South Africa; ^34^ Florey Institute of Neuroscience and Mental Health, Austin Campus Heidelberg Victoria Australia; ^35^ University of Melbourne Parkville Victoria Australia; ^36^ Department of Neurology Austin Health Heidelberg Victoria Australia; ^37^ Imaging Genetics Center, Mark and Mary Stevens Neuroimaging and Informatics Institute, Keck School of Medicine University of Southern California Marina del Rey California USA; ^38^ Institute of Systems, Molecular and Integrative Biology University of Liverpool Liverpool UK; ^39^ Department of Psychiatry University of Maryland School of Medicine Baltimore Maryland USA; ^40^ Department of Clinical Neurophysiology University Hospital Göttingen Göttingen Germany; ^41^ Department of Neurology and Epileptology Hertie Institute for Clinical Brain Research, University of Tübingen Tübingen Germany; ^42^ Clinical Neurophysiology University Medical Center Göttingen Göttingen Germany; ^43^ Pediatric Neurology, Neurogenetics and Neurobiology Unit and Laboratories A. Meyer Children's Hospital, University of Florence Florence Italy; ^44^ Functional and Epilepsy Neurosurgery Unit, Neurosurgery Department A. Meyer Children's Hospital, University of Florence Florence Italy; ^45^ Department of Neurology Royal Melbourne Hospital Melbourne Victoria Australia; ^46^ Department of Medicine, Royal Melbourne Hospital University of Melbourne Parkville Victoria Australia; ^47^ Mario Serio Department of Clinical and Experimental Medical Sciences University of Florence Florence Italy; ^48^ Psychiatric Genetics QIMR Berghofer Medical Research Institute Brisbane Queensland Australia; ^49^ Department of Biomedical, Metabolic, and Neural Sciences University of Modena and Reggio Emilia Modena Italy; ^50^ Neurology Unit, OCB Hospital Modena University Hospital Modena Italy; ^51^ Department of Neurology University of Campinas Campinas Brazil; ^52^ Cleveland Clinic Neurological Institute Cleveland Ohio USA; ^53^ School of Computing Newcastle University Newcastle Upon Tyne UK; ^54^ Division of Neuroscience King's College London London UK; ^55^ Giannina Gaslini Institute, Scientific Institute for Research and Health Care Genoa Italy; ^56^ Department of Neurosciences, Rehabilitation, Ophthalmology, Genetics, Maternal and Child Health University of Genoa Genoa Italy; ^57^ Department of Epileptology University Hospital Bonn Bonn Germany; ^58^ Department of Neuroscience, Central Clinical School, Alfred Hospital Monash University Melbourne Victoria Australia; ^59^ Departments of Medicine and Radiology, Royal Melbourne Hospital University of Melbourne Parkville Victoria Australia; ^60^ School of Electrical and Computer Engineering College of Engineering, University of Tehran Tehran Iran; ^61^ SA MRC Unit on Risk and Resilience in Mental Disorders, Department of Psychiatry and Neuroscience Institute University of Cape Town Cape Town South Africa; ^62^ Primary Care Department Local Health Authority of Modena Modena Italy; ^63^ Institute of Experimental Epileptology and Cognition Research University of Bonn Bonn Germany; ^64^ Support Center for Advanced Neuroimaging University Institute of Diagnostic and Interventional Neuroradiology, Inselspital, Bern University Hospital, University of Bern Bern Switzerland; ^65^ Department of Medicine, Division of Neurology Queen's University Kingston Ontario Canada

**Keywords:** disease progression, duration of illness, event‐based model, MTLE, patient staging

## Abstract

**Objective:**

Recent work has shown that people with common epilepsies have characteristic patterns of cortical thinning, and that these changes may be progressive over time. Leveraging a large multicenter cross‐sectional cohort, we investigated whether regional morphometric changes occur in a sequential manner, and whether these changes in people with mesial temporal lobe epilepsy and hippocampal sclerosis (MTLE‐HS) correlate with clinical features.

**Methods:**

We extracted regional measures of cortical thickness, surface area, and subcortical brain volumes from T1‐weighted (T1W) magnetic resonance imaging (MRI) scans collected by the ENIGMA‐Epilepsy consortium, comprising 804 people with MTLE‐HS and 1625 healthy controls from 25 centers. Features with a moderate case–control effect size (Cohen *d* ≥ .5) were used to train an event‐based model (EBM), which estimates a sequence of disease‐specific biomarker changes from cross‐sectional data and assigns a biomarker‐based fine‐grained disease stage to individual patients. We tested for associations between EBM disease stage and duration of epilepsy, age at onset, and antiseizure medicine (ASM) resistance.

**Results:**

In MTLE‐HS, decrease in ipsilateral hippocampal volume along with increased asymmetry in hippocampal volume was followed by reduced thickness in neocortical regions, reduction in ipsilateral thalamus volume, and finally, increase in ipsilateral lateral ventricle volume. EBM stage was correlated with duration of illness (Spearman *ρ =* .293, *p* = 7.03 × 10^−16^), age at onset (*ρ =* −.18, *p =* 9.82 × 10^−7^), and ASM resistance (area under the curve = .59, *p* = .043, Mann–Whitney *U* test). However, associations were driven by cases assigned to EBM Stage 0, which represents MTLE‐HS with mild or nondetectable abnormality on T1W MRI.

**Significance:**

From cross‐sectional MRI, we reconstructed a disease progression model that highlights a sequence of MRI changes that aligns with previous longitudinal studies. This model could be used to stage MTLE‐HS subjects in other cohorts and help establish connections between imaging‐based progression staging and clinical features.

**Key Points**

- We estimated the sequence of progression of subcortical and neocortical atrophy in MTLE with HS
- Abnormality started in the hippocampus, followed by decreased cortical thickness in the parietal and frontal lobes, thalamic volume, and ventricular expansion
- Image‐based disease stages were correlated with duration of illness, age at onset, and drug resistance
- Associations were driven by MTLE‐HS cases showing mild volume loss in the ipsilateral hippocampus that was indistinguishable from variation in the control group

## 1 INTRODUCTION

Epilepsy is characterized by recurrent seizures caused by excessive and abnormal neuronal activity in the cortex. Moreover, there is consistent evidence indicating decreased gray matter volume in people with epilepsy (PWE) compared to healthy controls. Quantitative analysis of MRI data from PWE in a large multicenter cohort showed reduced cortical thickness and subcortical volume in specific brain regions according to epilepsy type.^1^ In people with focal epilepsy, differences tend to be more pronounced ipsilateral to the seizure focus.^1^, ^2^ Beyond cortical thickness and subcortical volume differences, surface area reduction in the mesial and anterior temporal cortex has been previously reported.^3^

Whether seizures, antiseizure medication (ASM), head injuries, the epileptogenic process, the maintenance of seizure occurrence, or other comorbidities cause the observed loss of brain tissue is a much‐discussed question. Many studies have found that gray matter thickness is correlated with the duration of illness in the common epilepsies, indicating that these cross‐sectional differences may be progressive.^1^, ^4^, ^5^, ^6^, ^7^ Deciphering how gray matter reductions unfold over time in epilepsy is of great importance, but progress has been limited by the scarcity of longitudinal imaging cohorts. Recent work in this field has leveraged advanced mathematical models to infer longitudinal atrophy patterns from cross‐sectional data. For instance, in a dataset of people with mesial temporal lobe epilepsy (MTLE), Zhang et al. used Granger causality analysis to determine whether a previously affected region, or a group of regions, helped to predict the next brain region to exhibit atrophy; they found that subcortical regions such as the hippocampus and thalamus causally affected other regions, most prominently the prefrontal cortex and cerebellum.^8^ This approach, however, does not allow direct inference of a temporal sequence. A major step toward addressing the question of progression was provided by previous longitudinal studies that assessed progressive atrophy in patients with TLE,^5^, ^7^, ^9^ and prior meta‐analytical studies on the topic.^10^ One recent study investigated people with focal epilepsy and longitudinal MRI scans at least 6 months apart, showing that the annualized rate of atrophy within brain regions structurally connected to the ipsilateral hippocampus exceeded the rate associated with healthy aging^11^; although they demonstrated the progressive nature of atrophy, their approach did not address whether there is an explicit sequence in which these structural changes occur or whether this sequence can be used to stage epilepsy. Moreover, lower hippocampal volume has been reported in nonaffected siblings and thus may reflect a genetic origin^12^, ^13^, ^14^, ^15^ predating any further changes such as cortical thinning, which was not observed in siblings.^16^ We surmise that staging epilepsy in patients using a single MRI scan will help future research to assess the effectiveness of ASMs and disease‐modifying agents, for example, by directly establishing a link between disease stage and drug response or by improving efficacy of inclusion criteria for clinical trials of ASM candidates. Furthermore, understanding the spatial progression of atrophy in MTLE could help answer questions such as whether unilateral MTLE with hippocampal sclerosis (HS) can lead to bilateral HS in an individual patient.

In this work, we investigated disease progression in patients with radiographically identified sclerosis of the hippocampus or the mesial temporal lobe (MTLE‐HS) using the event‐based model (EBM). In brief, the EBM is a machine learning approach that learns the most likely ordering of biomarker changes from cross‐sectional data. The EBM was originally developed to study progressive loss of brain tissue in Alzheimer and Huntington diseases.^17^ A trained EBM can be used to assign a disease stage to each patient based on their atrophy pattern.^18^ Since it was introduced, the EBM has been used across a wide range of neurological diseases, including multiple sclerosis,^19^, ^20^ amyotrophic lateral sclerosis,^21^ and Parkinson disease.^22^ By applying the EBM to cross‐sectional data from PWE, we aimed to answer two questions. First, is there a characteristic order in which regional brain MRI morphometric changes develop in MTLE‐HS? Second, is the accumulation of imaging changes related to clinical markers of disease duration or severity?

## 2 MATERIALS AND METHODS

### 2.1 Data

We analyzed data from the ENIGMA‐Epilepsy working group^23^ comprising imaging data from controls and people with epilepsy from 25 centers (Table 1). Each center received approval from its local institutional review board or ethics committee. Written informed consent was provided according to local requirements. As previously described,^1^ T1‐weighted (T1W) brain MRI scans were acquired using 1.5‐T or 3‐T MRI scanners from different manufacturers and different imaging sequences. Brain scans were processed at each contributing center using the same pipeline based on FreeSurfer version 5.3.0.^24^, ^25^ Diagnosis of left and right MTLE was made by an epilepsy specialist at each center, based on seizure semiology and electroencephalographic findings. Presumed sclerosis of the hippocampus or the mesial temporal lobe was diagnosed according to established features on MRI (i.e., a T2‐weighted or fluid‐attenuated inversion recovery scan). In some cases, HS was confirmed based on histology from resected tissue. A common set of 156 regional features was extracted based on the Desikan–Killiany atlas^26^: 68 measures of regional cortical thickness (CT), 68 measures of regional surface area (SA), two measures of hemispheric average CT, two measures of hemispheric SA, and 16 subcortical brain volumes as previously described in detail.^1^ Since the initial study,^1^ five new centers were added, providing an additional 244 subjects. Overall, the ENIGMA‐Epilepsy dataset features preprocessed MRI scans from 1625 controls as well as 446 left MTLE‐HS and 358 right MTLE‐HS patients. After segmentation quality assurance, certain regional brain measures were removed for some subjects in the acquired dataset (about .02% of the values). We removed subjects with >10 missing values (66 subjects). Missing measures in the remaining subjects were imputed within each center using a singular value decomposition‐based approach.^27^ Additionally, age, sex, case–control status, lateralization (left or right MTLE‐HS), age at onset, and duration of illness were available. Furthermore, drug‐resistance status (defined as one or more seizures in the 12 months before MRI) was obtained for 408 MTLE‐HS cases.

**TABLE 1:** Cohort overview

Center	Age of controls, years, mean ± SD	Age of cases, years, mean ± SD	Age at onset, years, mean ± SD	Duration of illness, years, mean ± SD	Female controls, *n*	Female cases, *n*	Total controls, *n*	Total cases, *n*	L MTLE‐HS cases, *n*	R MTLE‐HS cases, *n*	Total, *N*
Bern	32.5 ± 9.39	31.3 ± 9.09	N/A	N/A	41	9	78	18	10	8	96
Bonn	40.4 ± 13.79	40.2 ± 13.37	17.1 ± 12.14	23 ± 14.16	41	62	80	112	74	38	192
CUBRIC	28 ± 8.17	N/A	N/A	N/A	34	0	48	0	0	0	48
EKUT	35.3 ± 12.33	N/A	N/A	N/A	9	0	18	0	0	0	18
EPICZ	38.8 ± 11.08	39.7 ± 9.11	18.1 ± 14.15	21.6 ± 13.48	59	26	116	46	19	27	162
EPIGEN_3T	34.7 ± 9.37	40.4 ± 6.28	21.8 ± 13.16	18.5 ± 11.98	30	6	70	13	8	5	83
Florence	32.2 ± 8.84	N/A	N/A	N/A	14	0	30	0	0	0	30
Genoa	25.2 ± 8.23	N/A	N/A	N/A	8	1	20	1	0	1	21
Greifswald	26.3 ± 7.48	N/A	N/A	N/A	59	0	99	0	0	0	99
HFHS	N/A	40.4 ± 14.85	10.4 ± 12.96	25.4 ± 14.44	0	15	0	20	9	11	20
IDIBAPS	33.1 ± 5.99	37.4 ± 9.94	17.7 ± 12.79	18.8 ± 9.97	29	29	52	53	17	36	105
KCL_CNS	31.7 ± 8.4	41 ± 9.57	17.5 ± 14.16	25.2 ± 16.97	54	11	101	15	6	9	116
KCL_CRF	28.7 ± 8.29	37.8 ± 11.52	22.6 ± 12.34	15.2 ± 8.04	16	1	26	5	3	2	31
KUOPIO	25.2 ± 1.55	41.1 ± 11.06	23.3 ± 18.23	17.8 ± 17.02	33	5	67	9	0	9	76
MICA	31.9 ± 4.77	38.9 ± 13.12	23.4 ± 11.71	15.7 ± 14.58	18	7	38	14	12	2	52
MNI	30.7 ± 7.38	33.6 ± 9.53	17.3 ± 10.57	16.3 ± 11.4	22	48	46	83	45	38	129
MUSC	54.9 ± 8.4	33.5 ± 12.73	15.4 ± 12.34	18.2 ± 12.79	45	17	58	27	21	6	85
NYU	30.1 ± 10.36	33.8 ± 9.31	14.1 ± 8.04	20.2 ± 14.44	62	12	118	19	8	11	137
RMH	38.8 ± 20.44	39.6 ± 15.59	27.1 ± 17.69	12.4 ± 13.23	11	13	27	35	22	13	62
UCL	37.7 ± 12.4	39.5 ± 11.29	11.8 ± 8.72	27.7 ± 15.12	17	21	29	37	24	13	66
UCSD	36.9 ± 15.1	39.2 ± 12.53	15.6 ± 12.44	24.3 ± 17.82	16	15	37	26	16	10	63
UMG	34.7 ± 10.26	40.6 ± 12.49	15.4 ± 14.04	23.9 ± 18.49	12	12	21	20	10	10	41
UNAM	33.2 ± 12.29	34.4 ± 12.47	15.5 ± 13.84	18.8 ± 13.16	25	12	35	20	10	10	55
UNICAMP	34.4 ± 10.47	42.7 ± 8.33	11.4 ± 9.6	31.3 ± 12.13	249	113	398	191	107	84	589
XMU	31.5 ± 7	28.2 ± 8.45	17.2 ± 12.06	11.3 ± 8.02	4	15	13	40	25	15	53
Total	33.8 ± 11.45	38.5 ± 11.44	15.9 ± 12.4	22.7 ± 14.39	908	450	1625	804	446	358	2429

*Note*: Individual cohort demographics include age, number of left and right MTLE‐HS, and controls, as well as age at onset and duration of illness for MTLE‐HS patients.

Abbreviations: L, left; MTLE‐HS, mesial temporal lobe epilepsy with hippocampal sclerosis; N/A, not available; R, right.

### 2.2 Data harmonization and confound adjustment

Because ENIGMA‐Epilepsy is a multicentric study, the data are subject to center‐specific biases arising from various factors. Thus, all 156 regional brain measures were harmonized for center biases using NeuroCombat,^28^, ^29^ while retaining variation originating from age, sex, intracranial volume (ICV), and diagnosis. Following the harmonization, the regional measures were adjusted for ICV, age, and sex using linear regression. As in previous work,^30^ the residuals for each regional measure plus the intercept of the model were used as confound‐adjusted measures for the remaining analysis.

### 2.3 Ipsilateral and contralateral features

Studies have shown unilateral and bilateral alterations of structural connectivity and structural measures in left and right MTLE‐HS patients, with the ipsilateral regions being more strongly affected.^30^, ^31^, ^32^, ^33^ To estimate a progression pattern for MTLE‐HS regardless of lateralization, we jointly analyzed left and right MTLE‐HS cases. Therefore, we replaced "left" and "right" with "ipsilateral" and "contralateral" (e.g., left hemisphere is ipsilateral in left MTLE‐HS and contralateral in right MTLE‐HS). For the controls, we randomly sampled half as controls for left MTLE‐HS, where left and right hemispheres were defined as ipsilateral and contralateral regions, respectively. Similarly, the remaining half acted as controls for right MTLE‐HS with the hemispheres swapped. Overall, this enabled us to study brain regions commonly affected in both left and right MTLE‐HS.

### 2.4 Brain asymmetry index features

Previous studies^2^, ^34^ have used the asymmetry of brain regions to model atrophy in people with MTLE‐HS. The rationale is that contralateral brain regions of each subject act as a personalized healthy reference region (in cases where pathology manifests unilaterally) and therefore may act as an earlier, more sensitive marker, in the EBM. We computed the brain asymmetry index (BASI) for regional cortical thickness, surface area, and volume as the following ratio:

$$\text{BASI}=\frac{\left(\text{ipsilateral}–\text{contralateral}\right)}{\left(\text{ipsilateral}+\text{contralateral}\right)/2}$$

### 2.5 Feature selection

First, we sought to identify brain regions with sufficient epilepsy‐related atrophy to be used for progression modeling. We used a robust variant of Cohen *d* ^35^ between MTLE‐HS cases and controls for all 234 features (78 ipsilateral, 78 contralateral, and 78 BASI). Robust Cohen *d* uses the median and mean absolute deviation in place of the mean and SD, respectively, and is more resilient against outliers.^36^ A medium effect size (robust Cohen |*d*| ≥ .5) was required for inclusion into disease progression modeling. We also evaluated a more lenient threshold (robust Cohen |*d*| *≥* .4).

### 2.6 Event‐based modeling

**FIGURE 1:**
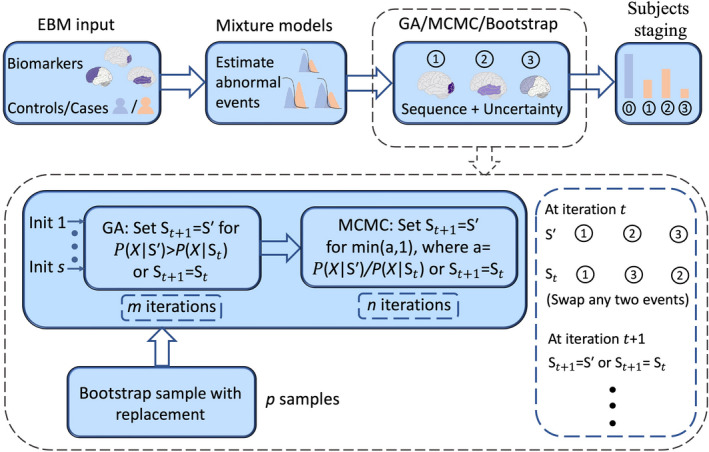
Event‐based model (EBM) workflow. A set of *k* biomarkers and case–control status are provided for each subject. Then, mixture modeling is used to estimate distributions of the biomarkers in cases and controls, respectively. The maximum likelihood sequence (i.e., optimal ordering) of the *k* biomarkers is estimated using Markov chain Monte Carlo (MCMC) with 500 000 iterations. The MCMC sequence is initialized using 10 random starting solutions and a greedy ascent (GA) run for 10 000 iterations. Finally, in a third step, we used 100 bootstrap samples to determine the uncertainty and variability of the sequence. Init, initialization.

The selected regions were used as inputs to the EBM.^17^ The EBM relies on two main assumptions: (1) biomarkers become abnormal sequentially; and (2) biomarkers follow a monotonic trajectory during disease progression, where an abnormal marker will not revert to a normal stage. Thus, the model assumes that for any given cross‐sectional dataset, a greater proportion of patients will show abnormalities for early stage biomarkers, whereas fewer patients will also have abnormal later stage biomarkers. Furthermore, the model requires distributions that define what normal and disease‐specific measures look like for every biomarker. In practice, an overlap between the normal and disease‐specific distributions for biomarkers is expected. We used a kernel density estimation‐based (KDE)^37^ mixture model that provides estimations of case and control distributions even when they are skewed or do not follow a parametric distribution.^38^ Next, the EBM determines the most likely ordering of biomarkers for the given dataset, as illustrated in Figure 1. Practically, the ordering is obtained using a maximum likelihood approach. Greedy ascent is used to initialize the sequence estimation, and Markov chain Monte Carlo (MCMC) sampling is used to perform the maximum likelihood estimation. The MCMC samples are used to derive a characteristic ordering of the events along with its variability. We used 10 000 iterations per chain during the greedy ascent initialization and generated 500 000 MCMC samples. Finally, to generate a conservative, upper‐bound estimate of the variability of the sequence, we combined the sequence estimation with bootstrapping (100 repeats) and generated positional variance diagrams from these bootstraps. The patient staging mechanism^18^ is then used to assign each of the control subjects and MTLE‐HS cases to a disease stage ranging from 0 (i.e., no abnormality) to an asymptotic endpoint, which equals the number of biomarkers selected for analysis (i.e., all biomarkers abnormal). To investigate whether the biomarker sequence is consistent in cases with left and right MTLE‐HS, we trained EBMs for these two groups separately.

### 2.7 Association of EBM stages with duration of illness, age at onset, and treatment response

We hypothesized that subjects with advanced EBM stages were more likely to have a longer duration of illness, to have earlier disease onset, and to be drug‐resistant. To determine whether individuals' EBM stage is related to illness duration or age at onset, we computed Spearman rank correlations between EBM stage and the duration of illness (in years) at the time of imaging and age at onset, respectively. Furthermore, we used the Mann–Whitney *U* test to test for a difference in EBM‐assigned stage regarding drug‐resistant status.

## 3 RESULTS

Table 1 displays the overall cohort split by center. On average, each center contributed a range of individuals, ranging from young adults in their 20s to adults over 60 years old (median = 33.0 years, interquartile range [IQR] = 18.08 years). The binary sex distribution within the dataset was well balanced, with a slight majority of women (56.0% of MTLE‐HS patients and 55.9% of healthy controls). The duration of illness ranged from recently diagnosed to 68 years (median = 20.0 years, IQR = 24.0 years).

### 3.1 Effect sizes of selected features

**FIGURE 2:**
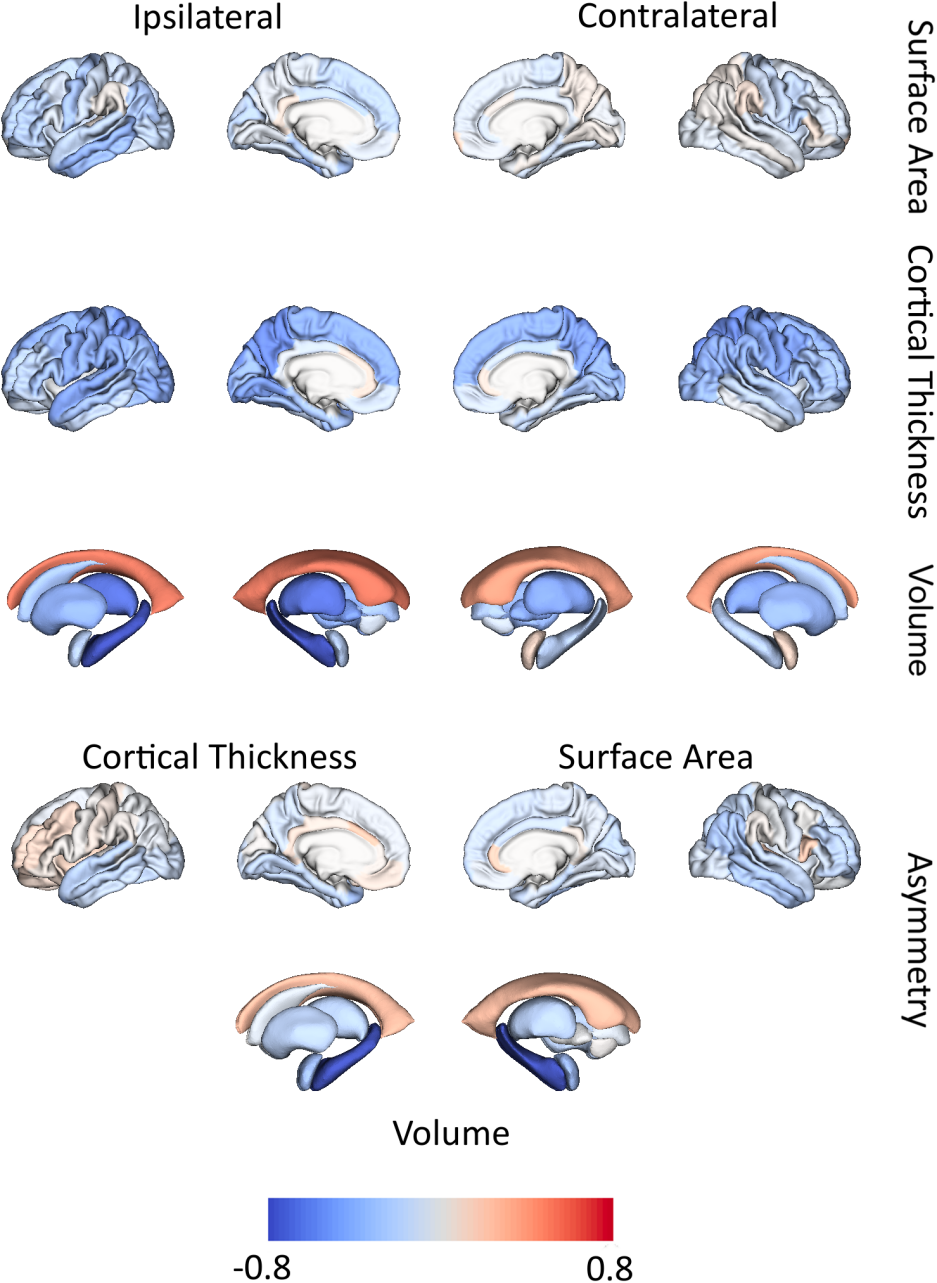
Regional differences in sclerosis of hippocampus or mesial temporal lobe (MTLE‐HS) compared to controls. Effect sizes between MTLE‐HS cases and controls measured as *robust Cohen d* for surface area, cortical thickness, and volume are depicted ipsilateral or contralateral to the seizure focus (top three rows). The bottom two rows depict effect sizes for asymmetry features.

The seven selected features (robust Cohen |*d*| ≥ .5) were ipsilateral hippocampal volume and its BASI, ipsilateral thalamic volume, cortical thickness of bilateral superior parietal gyrus, and ipsilateral precuneus and ipsilateral lateral ventricle volume (Table S1). Figure 2 provides a visual representation of the effect sizes rendered using the ENIGMA toolbox.^39^ Our mega‐analysis replicated the finding of the original ENIGMA‐Epilepsy meta‐analysis.^1^ Effect sizes (robust Cohen *d*) ipsilateral to the seizure focus were stronger than those in the corresponding contralateral region for the surface area (*t* = 4.01, *p* = .00033, *df* = 33, paired *t*‐test) but not for cortical thickness (*t* = 1.95, *p* = .06, *df* = 33, paired *t*‐test) nor for subcortical volumes (*t* = 1.60, *p* = .15, *df* = 7, paired *t*‐test). Effect sizes for cortical thickness were stronger than effect sizes for surface area (*t* = 8.08, *p* = 1.09 × 10^−11^, *df* = 67, paired *t*‐test). Use of the lower Cohen *d* cutoff of .4 produced 12 additional features for EBM modeling (Table S1).

### 3.2 Sequence of abnormal biomarkers in left and right MTLE‐HS

**FIGURE 3:**
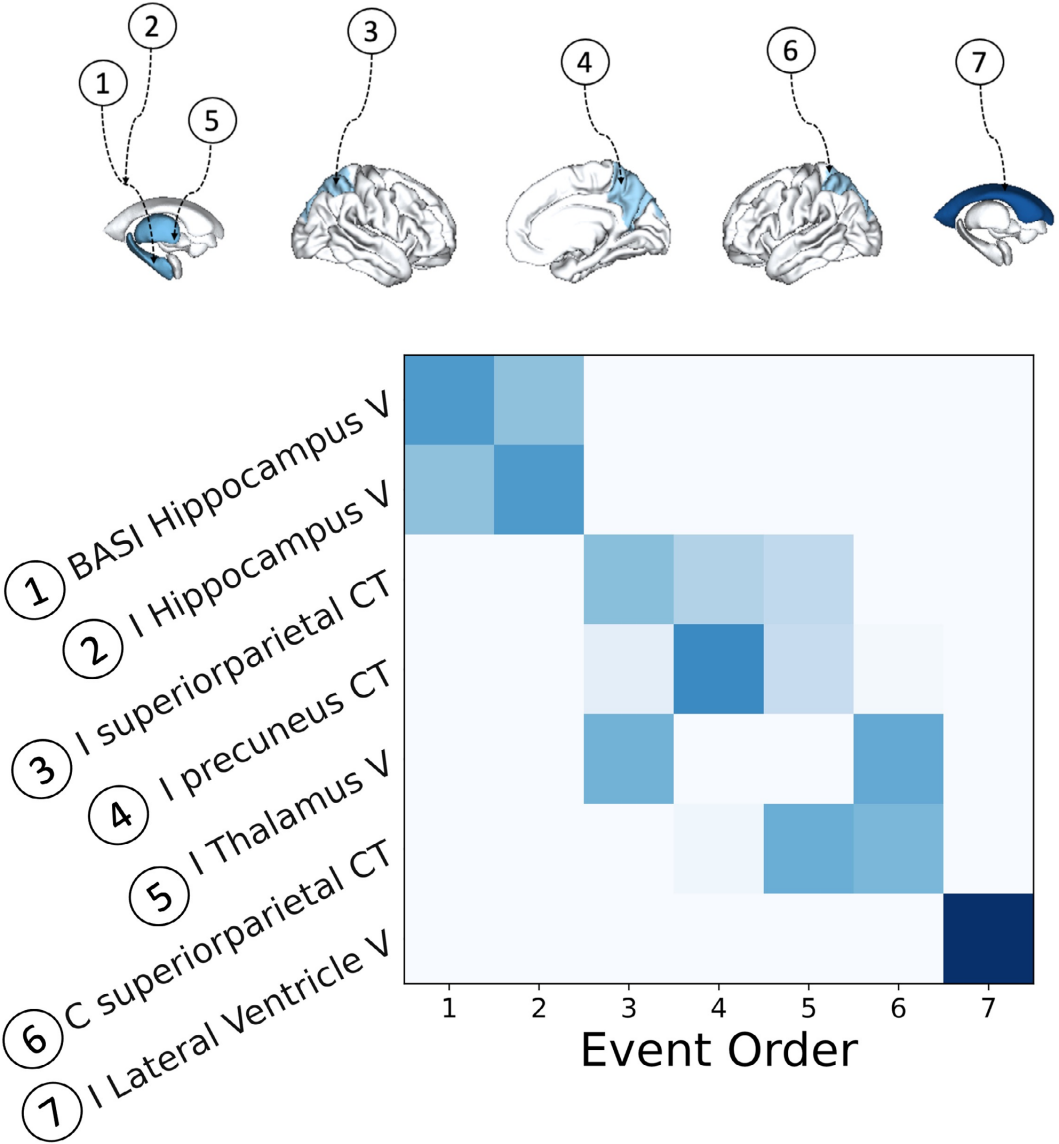
Sequential accumulation of pathology in sclerosis of hippocampus or mesial temporal lobe. Data‐driven sequence of atrophy or increased asymmetry of brain regions is shown. Color intensity in the positional variance diagram (PVD) the proportion of certainty (.0 in white to 1.0 in dark blue) in which biomarkers (*y*‐axis) appear in a particular position (*x*‐axis) in the event order obtained through bootstrapping. BASI, brain asymmetry index; C, contralateral; CT, cortical thickness; I, ipsilateral; V, volume.

The EBM estimated the sequence for the seven selected imaging biomarkers using the KDE mixture models (Figure S1) and placed them in Stages 0 to 7 (Figure 3). The bootstrapped version of the EBM placed reduced ipsilateral hippocampal volume and increased asymmetry in hippocampal volume at the beginning of the sequence. This was followed by decreased cortical thickness and decreased ipsilateral thalamic volume (Figure 3). We analyzed left and right MTLE‐HS cases separately, with similar progression patterns in both syndromes (Figure S2). Reducing the inclusion threshold to Cohen |*d*| ≥ .4 led to 19 biomarkers and provided a more fine‐grained staging, but with essentially the same progression sequence as in the original analysis (Figure S3).

### 3.3 Cross‐sectional distribution of patients across disease stages as defined by EBM

**FIGURE 4:**
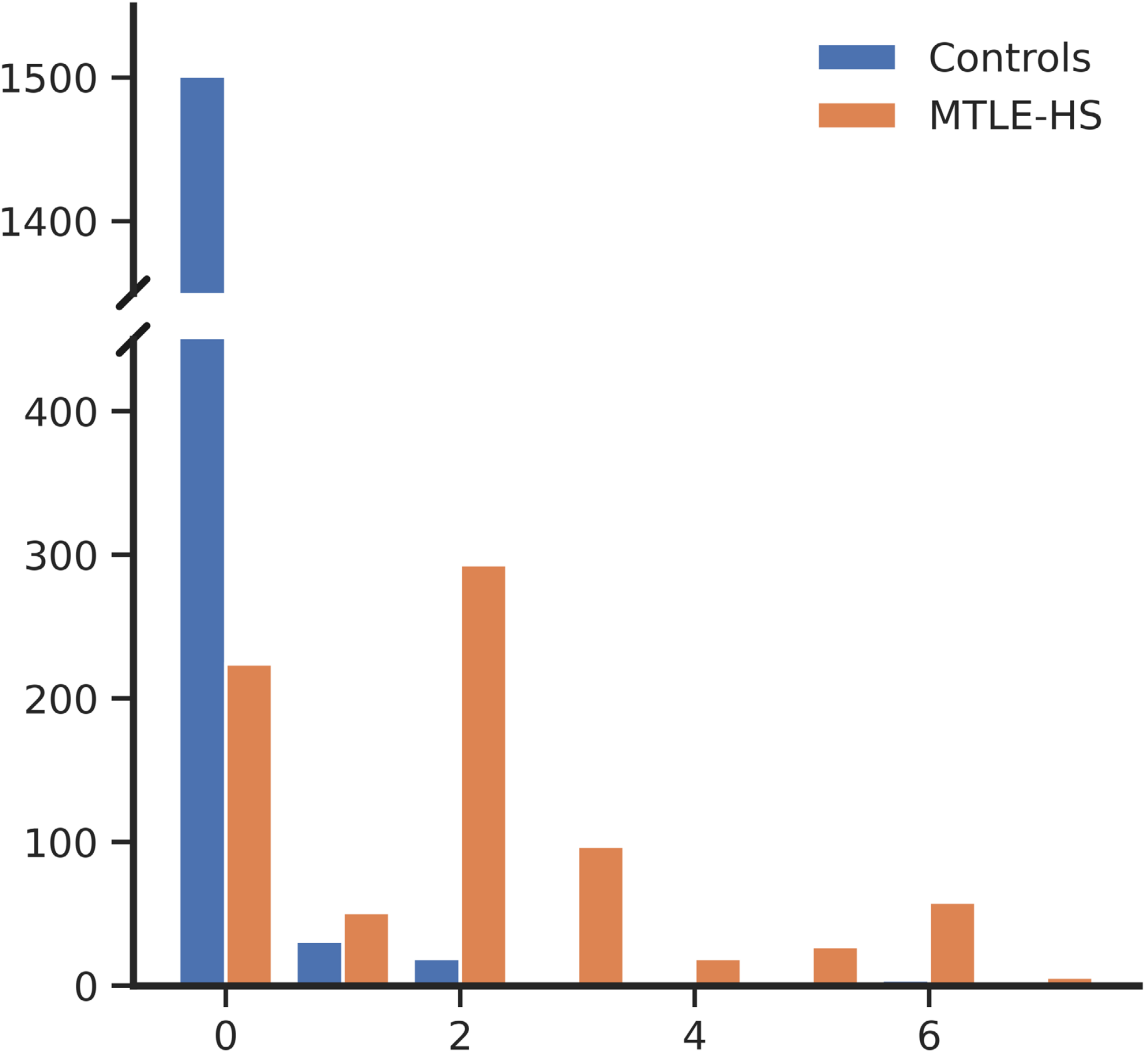
Event‐based model (EBM) stage distribution. Histogram shows stages (*x*‐axis) assigned to controls and people with mesial temporal lobe epilepsy and hippocampal sclerosis (MTLE‐HS) and the corresponding count (*y*‐axis). Stage 0 is assigned to subjects with no statistically detectable abnormal brain region based on the T1‐weighted magnetic resonance imaging scans. EBM places subjects with abnormal features progressively, such that subjects in Stage 7 exhibit abnormality in all seven regional measures.

We used the trained EBM to stage participants based on brain regions with structural alterations^18^; controls and PWE were assigned to Stages 0–7. Most of the MTLE‐HS cases (71.1%) were staged at Stage 1 or greater (Figure 4). However, a large proportion of MTLE‐HS cases (28.9%) were staged at 0, indicating mild or nondetectable abnormality on T1W MRI. Approximately 44.4% were assigned to Stages 1 and 2, reflecting reduced volume of the ipsilateral hippocampus and abnormal asymmetry in the hippocampus. The remaining MTLE‐HS cases (26.7%) were staged beyond Stage 2, suggesting neocortical involvement, reduction of ipsilateral thalamic volume, and increase in ipsilateral lateral ventricle volume. The distribution of stages differed between left and right MTLE‐HS cases (*H* = 7.35, *p* = .0067, Kruskal–Wallis test; Figure S4).

Ipsilateral hippocampal volumes in cases at Stage 0 were significantly larger than in cases assigned to later stages (*t* = 32.35, *p* = 7.77 × 10^−146^, *t*‐test; Figure S5). Consequently, effect size of ipsilateral hippocampal volume was *d* = −.31 and *d* = −2.09 for cases at Stage 0 and non‐0 stages, respectively. In addition, cases assigned to EBM Stages 3–7 exhibited reduced contralateral hippocampal volume compared to controls (*d* = −.54), which was not observed in cases assigned to Stage 0 (*d* = −.17) or Stages 1–2 (*d* = .16).

### 3.4 EBM stage is associated with duration of illness and with response to ASMs in MTLE patients

**FIGURE 5:**
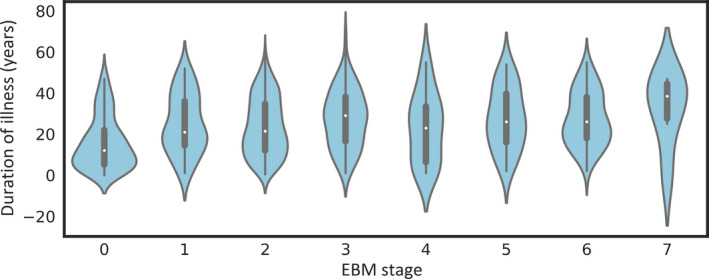
Distribution of duration of illness per event‐based model (EBM) stage. Violin plots showing distribution of duration of illness (in years) of corresponding EBM Stages 0–7 of sclerosis of hippocampus or mesial temporal lobe (MTLE‐HS) patients. MTLE‐HS cases assigned to EBM Stage 0 showed a shorter duration of illness compared to cases assigned to the remaining EBM stages.

MTLE‐HS patients assigned to early EBM stages showed a relatively shorter illness duration than those in later stages (Figure 5). Duration of illness and Stages 0–7 were significantly correlated in all MTLE‐HS cases (Spearman *ρ* = .293, *p* = 7.03 × 10^−16^). After excluding cases at Stage 0, the correlation remained marginally significant (Spearman *ρ* = .099, *p* = .024). Thus, the correlation is driven by the significant difference in duration of illness between EBM Stage 0 (mean = 15.7 years) and non‐0 (mean = 25.1 years, *t* = −8.23, *p* = 8.63 × 10^−16^). The same pattern was observed for age at onset; EBM stage and age at onset were negatively correlated (*ρ* = −.18, *p* = 9.82 × 10^−7^), but the effect vanished in the subset of cases at Stages 1–7 (*ρ* = .004, *p* = .92). Age at onset was significantly later for Stage 0 cases compared to non‐0 cases (*t* = 5.69, *p* = 1.75 × 10^−8^). EBM stages differed between MTLE‐HS cases that were resistant (*n* = 363) or responsive (*n* = 45) to ASMs in the 12 months prior to MRI (area under the curve = .589, *p* = .043, Mann–Whitney *U* test).

## 4 DISCUSSION

We applied data‐driven disease progression modeling to a large, multicenter imaging study of epilepsy to characterize the progression of MTLE‐HS. We identified a characteristic order of MRI morphometric changes originating in the ipsilateral hippocampus. We did not identify statistically significant correlations between the accumulation of imaging changes (EBM Stages 1–7) and available clinical markers of disease duration or severity in this cohort.

For the progression modeling, we retained features exhibiting a medium effect size between cases and controls (|*d*| ≥ .5; |*d*| ≥ .4 for a sensitivity analysis; Table S1). Our most interesting observation was a pattern of brain atrophy that appears to progress from the ipsilateral hippocampus to bilateral neocortical regions (e.g., precuneus and superior parietal lobule) as well as the bilateral thalamus (Figure S3). Volume reduction and increased asymmetry in the hippocampus may represent a genetic predisposition to HS, because hippocampal abnormalities have frequently been observed in healthy siblings of people with MTLE,^12^, ^13^, ^14^, ^15^ and an association was observed in a genome‐wide association study.^40^ However, cortical thinning likely represents disease‐related effects, because these changes have not been reported in healthy siblings.^16^ Furthermore, the progression pattern included decline in thalamic volume, which is a common feature in MTLE‐HS^41^, ^42^, ^43^, ^44^ and may be linked to the strong structural connectivity between the hippocampus and the thalamus.^42^, ^45^, ^46^

At first glance, it appears surprising that many MTLE‐HS cases were assigned to Stage 0 despite the loss of hippocampal volume being one of the hallmark signs of MTLE‐HS. Two factors contribute to this discrepancy. First, the radiologic diagnosis of HS is based on multiple imaging sequences, whereas hippocampal atrophy, as defined on T1W images, is only one component of HS.^47^ Second, although we observed a large group effect size for hippocampal volume difference in the whole cohort (*d* = −1.76), there is significant variability in volume loss at the individual level. Approximately half the subjects with HS exhibit hippocampal volume that is within the normal range^48^; this is also the case in the ENIGMA‐Epilepsy cohort (Figures S1 and S1).

Duration of illness is typically used as a proxy for progression in cross‐sectional studies.^5^, ^10^, ^49^ Moreover, within ENIGMA‐Epilepsy,^1^ changes in numerous neocortical regions, subcortical volumes, and hippocampal volume were negatively correlated with duration of illness. However, these results were driven by epilepsies without HS; no correlations within the left MTLE‐HS subgroup were found to be statistically significant, and within the right MTLE‐HS group significant correlations were limited to the ipsilateral hippocampus, putamen, thalamus, contralateral transverse temporal gyrus, and ipsilateral caudal middle frontal gyrus. Therefore, the marginal correlations between EBM Stages 1–7 and duration of illness in subjects with MTLE‐HS agree with these earlier observations. Furthermore, Zhang et al.^8^ reported that measures of the ipsilateral hippocampus, the bilateral frontal lobes, and cerebellar hemispheres negatively correlated with duration of illness. However, in the same study, the lifetime number of seizures, another proxy for disease severity, was investigated and was correlated with atrophy in a different set of brain regions. Thus, either measure may capture different aspects of disease severity, and the relationship between disease duration and atrophy may be more complex. Disease duration and the other measures examined here are the most obvious and plausible factors to examine, and those most available, but may not be those that most influence the EBM‐derived sequence of changes we detect.

Longitudinal studies of PWE reveal cortical atrophy beyond the expected range of normal aging.^5^, ^7^, ^50^, ^51^ Moreover, recent longitudinal studies of people with focal epilepsy^11^, ^52^ found progressive atrophy in the contralateral regions of the parietal and frontal lobes, which was also featured in our study when using the more lenient cutoff (Figure S3). Overall, we find that our regional disease progression sequence, which is based on cross‐sectional data, agrees with previous findings in longitudinal cohorts that show the progressive nature of atrophy in MTLE‐HS.^5^, ^9^, ^10^ Contralateral hippocampal volume (*d* = −.14) missed the inclusion threshold for the EBM. Thus, the analysis could not provide further insights on whether untreated unilateral HS will lead to bilateral HS. However, PWE assigned to later EBM stages did present with reduced volume in the contralateral hippocampus, whereas this was not the case for PWE assigned to earlier stages, illustrating the potential of EBM.

The staging of individual MTLE‐HS patients using the trained EBM allowed us to investigate associations with duration of illness and clinical markers such as ASM resistance. In agreement with Whelan et al.^1^ and Zhang et al.,^8^ EBM‐based stages (Stages 0–7) and duration of illness were found to be correlated. However, this association was mainly driven by patients who were assigned to Stage 0. MTLE‐HS cases assigned to EBM Stage 0 did not show pronounced changes in ipsilateral hippocampal volume compared to controls (Figure S5) and as a group had shorter duration of illness and later age at onset than the other MTLE‐HS cases. Of note, the fraction of Stage 0 MTLE‐HS varied across centers (Figure S6) and may reflect differences between regional practices and capabilities to detect and diagnose mesial temporal sclerosis or HS.

There were several limitations in our study. First, this ENIGMA‐Epilepsy cohort is not a population‐based cohort but represents data mostly from tertiary epilepsy centers, and therefore the findings may not be generalizable to the overall epilepsy population. Also, within the ENIGMA‐Epilepsy cohort, we observed sampling bias regarding availability of ASM response data (Table S2); PWE with missing response data were younger, diagnosed more recently, and had later age at onset. Second, although the results were robust under bootstrap validation, they would benefit from validation in a longitudinal cohort. However, designing well‐powered longitudinal studies in controls and patients is challenging, especially because drug‐resistant TLE patients may eventually undergo epilepsy surgery.^10^ Third, clinical features such as lifelong ASM exposure were not available in the ENIGMA‐Epilepsy dataset and would prove difficult to ascertain retrospectively but should be considered in future work. The use of specific ASMs may affect disease progression and, in some cases, even amplify tissue loss in epilepsy.^53^ Finally, our model could be improved by considering measures from diffusion MRI scans to understand the role of white matter abnormalities in disease progression.^23^, ^54^

In conclusion, we estimated a sequence of progressive pathology in MTLE‐HS that can be used to assign patients to fine‐grained, image‐based disease stages. Beyond Stage 0, the EBM staging did not correlate with duration of illness, age at onset, or drug‐resistance. However, our EBM model trained on the ENIGMA‐Epilepsy data can be used to stage MTLE‐HS subjects in other cohorts with relevant clinical data and help establish connections between imaging‐based progression staging and other clinical features such as the lifetime number of seizures and detailed information on ASM exposure.

## AUTHOR CONTRIBUTIONS

Cohort principal investigators: A.A., A.B., A.G., A.L., B.W., C.R.M., D.J.S., F.C., G.D.J., G.P.W., H.S.‐Z., K.H., L.Co., M.R., N.Ba., N.Be., N.D., N.K.F., P.S., R.G., R.W., S.M., S.M.S., S.S.K., T.J.O., T.R. Contributed to the editing of the manuscript: A.A., A.B., A.G., A.R., A.E.V., B.Ben., B.Ber., C.R.M., C.M., D.C.A., D.J.S., E.D.‐B., E.G., E.K., E.L., F.C., G.D.J., G.P.W., J.S.D., K.H., L.Ca., L.Co., L.M.A., L.V., M.A., M.G., M.M., N.Be., N.J., N.P.O., P.M.D., P.S., S.E.M., S.I.T., S.L., S.M.L., S.M.S., S.S.K., T.J.O., V.I.D., L.B., S.B.V., E.B. Imaging data collection: A.B., A.R., B.Ben., B.W., C.d.B., C.D.W., C.L.Y., E.C.‐B., E.D.B., E.K., E.L., F.C., F.F., G.P.W., J.R., K.H., L.Co., L.V., M.A., M.E.M.‐S., M.M., M.R., N.Be., O.D., P.M., P.M.D., P.S., R.G., R.H.T., S.A., S.S.K., Y.W., L.B., E.B. Imaging data analysis: A.C., B.A.K.K., B.C., B.S., C.d.B., C.D.W., C.L.Y., D.C.A., E.D.‐B., J.R., K.H., L.M.A., L.V., M.E.C., M.D., M.L., M.R., M.T., N.J., P.K., P.M., P.N.T., R.G., R.K., S.F., S.M.L., S.S.K., T.W.O., Y.W., S.B.V. Core analysis group: A.A., C.R.M., D.C.A., N.P.O., S.M.L., S.M.S. Core writing group: A.A., C.R.M., D.C.A., N.P.O., S.M.L., S.M.S. Patient recruitment, phenotyping: R.H.T. ENIGMA‐Central: P.M.T., S.I.T., N.J.

## CONFLICT OF INTERESTS

B.Ben. is cofounder of AIRAmed, a company that offers brain segmentation. C.D.W. is an employee of Biogen. D.J.S. has received research grants and/or consultancy honoraria from Lundbeck and Sun. K.H. has received honoraria and speaker fees from UCB, Eisai, and GW Pharma L.V. reports research funding from Biogen Australia, Life Molecular Imaging, and Eisai. N.K.F. has received honoraria from Arvelle, Bial, Eisai, Philips/EGI, and UCB. N.J. is MPI of a research grant from Biogen for work unrelated to the contents of this article. P.S. has received speaker fees and served on advisory boards for Biomarin, Zogenyx, GW and Pharmaceuticals; has received research funding from ENECTA, GW Pharmaceuticals, Kolfarma, and Eisai. P.M.T. has received a research grant from Biogen and was a paid consultant for Kairos Venture Capital for projects unrelated to this work. None of the other authors has any conflict of interest to disclose. We confirm that we have read the Journal's position on issues involved in ethical publication and affirm that this report is consistent with those guidelines.

## Supporting information


APPENDIX S1
