# Supplementary material for: Event‐based modeling in temporal lobe epilepsy demonstrates progressive atrophy from cross‐sectional data
Source: Epilepsia. 2022 Jun 25;63(8):2081–95. doi: 10.1111/epi.17316 (PMC9540015; doi:10.1111/epi.17316)
Supplement: Supplementary file 1 — APPENDIX S1 [file EPI-63-2081-s001.docx]

**Supplementary Material**

**Event-based modelling in temporal lobe epilepsy demonstrates progressive atrophy from cross-sectional data**

Seymour M. Lopez^1^, Leon M. Aksman^1,2^, Neil P. Oxtoby^3^, Sjoerd B. Vos^1,4^, Jun Rao^5^, Erik Kaestner^5^, Saud Alhusaini^6,7^, Marina Alvim^8^, Benjamin Bender^9^, Andrea Bernasconi^10^, Neda Bernasconi^10^, Boris Bernhardt^11^, Lorenzo Caciagli^11,12^, Benoit Caldairou^10^, Maria Eugenia Caligiuri^13^, Angels Calvet^14^, Fernando Cendes^8^, Luis Concha^15^, Estefania Conde-Blanco^16,17^, Esmaeil Davoodi-Bojd^18^, Christophe de Bézenac^19^, Norman Delanty^7,20^, Patricia M. Desmond^21^, Orrin Devinsky^22^, Martin Domin^23^, John S. Duncan^12,24^, Niels K. Focke^25^, Sonya Foley^26^, Francesco Fortunato^27^, Marian Galovic^12,28^, Antonio Gambardella^13,27^, Ezequiel Gleichgerrcht^29^, Renzo Guerrini^30^, Khalid Hamandi^26,31^, Victoria Ives-Deliperi^32^, Graeme D. Jackson^33–35^, Neda Jahanshad^36^, Simon S. Keller^37^, Peter Kochunov^38^, Raviteja Kotikalapudi^9,39,40^, Barbara A.K. Kreilkamp^37,41^, Angelo Labate^13,27^, Sara Larivière^11^, Matteo Lenge^42,43^, Elaine Lui^21^, Charles Malpas^44,45^, Pascal Martin^40^, Mario Mascalchi^46^, Sarah E. Medland^47^, Stefano Meletti^48,49^, Marcia E. Morita-Sherman^50,51^, Thomas W. Owen^52^, Mark Richardson^53^, Antonella Riva^54,55^, Theodor Rüber^56^, Ben Sinclair^57,58^, Hamid Soltanian-Zadeh^18,59^, Dan J. Stein^60^, Pasquale Striano^54,55^, Peter N. Taylor^12,52^, Sophia I. Thomopoulos^36^, Paul M. Thompson^36^, Manuela Tondelli^48,61^, Anna Elisabetta Vaudano^48,49^, Lucy Vivash^57,58^, Yujiang Wang^12,52^, Bernd Weber^62^, Christopher D. Whelan^7^, Roland Wiest^63^, Gavin P. Winston^12,24,64^, Clarissa Lin Yasuda^8^, Carrie R. McDonald^5^, Daniel C. Alexander^3^, Sanjay M. Sisodiya^12,24^, Andre Altmann^1^ for the ENIGMA-Epilepsy Working Group*

Consortium banner:

*Núria Bargalló^14,65,66^, Emanuele Bartolini^30^, Terence J. O'Brien^45,57^, Rhys H. Thomas^67^

Affiliations

1. Centre for Medical Image Computing, Department of Medical Physics and Biomedical Engineering, University College London, London, UK.

2. Stevens Neuroimaging and Informatics Institute, Keck School of Medicine, University of Southern California, Los Angeles, CA, USA.

3. Centre for Medical Image Computing, Department of Computer Science, University College London, London, UK.

4. Neuroradiological Academic Unit, UCL Queen Square Institute of Neurology, University College London, London, UK.

5. Department of Psychiatry, University of California San Diego, La Jolla, CA, USA.

6. Department of Neurology, Alpert Medical School of Brown University, Providence, RI, USA.

7. Department of Molecular and Cellular Therapeutics, The Royal College of Surgeons in Ireland, Dublin, Ireland.

8. Department of Neurology and Neuroimaging Laboratory, University of Campinas - UNICAMP, Campinas, SP, Brazil.

9. Department of Radiology, Diagnostic and Interventional Neuroradiology, University Hospital Tübingen, Tübingen, Germany.

10. Neuroimaging of Epilepsy Laboratory, Montreal Neurological Institute, McGill University, Montreal, QC, Canada.

11. Multimodal Imaging and Connectome Analysis Laboratory, McConnell Brain Imaging Centre, Montreal Neurological Institute and Hospital, McGill University, Montreal, QC, Canada.

12. Department of Clinical and Experimental Epilepsy, UCL Queen Square Institute of Neurology, London, WC1N 3BG, UK.

13. Neuroscience Research Center, Department of Medical and Surgical Sciences, University “Magna Græcia" of Catanzaro, Catanzaro, Italy.

14. Magnetic Resonance Image Core Facility, Institut d’Investigacions Biomèdiques August Pi i Sunyer (IDIBAPS), Universitat de Barcelona, Barcelona, Spain.

15. Instituto de Neurobiología, Universidad Nacional Autónoma de México, Querétaro, Mexico.

16. Epilepsy Program, Neurology Department, Hospital Clínic de Barcelona, Barcelona, Spain.

17. Institut D’Investigacions Biomèdiques August Pi i Sunyer (IDIBAPS), Barcelona, Spain.

18. Radiology and Research Administration, Henry Ford Health System, Detroit, MI, USA.

19. Department of Pharmacology and Therapeutics, Institute of Systems, Molecular and Integrative Biology, University of Liverpool, Liverpool, UK.

20. FutureNeuro SFI Research Centre for Rare and Chronic Neurological Diseases, Dublin, Ireland.

21. Department of Radiology, The Royal Melbourne Hospital, University of Melbourne, Melbourne, VIC, Australia.

22. New York University Grossman School of Medicine, New York City, NY, USA.

23. Functional Imaging Unit, Department of Diagnostic Radiology and Neuroradiology, University Medicine Greifswald, Greifswald, Germany.

24. Chalfont Centre for Epilepsy, Chalfont St Peter, Bucks, UK.

25. Department of Neurology, University Medical Center, Göttingen, Germany.

26. Cardiff University Brain Research Imaging Centre, School of Psychology, Cardiff University, Cardiff, UK.

27. Institute of Neurology, Department of Medical and Surgical Sciences, University “Magna Græcia" of Catanzaro, Catanzaro, Italy.

28. Department of Neurology, University Hospital Zurich, Zurich, Switzerland.

29. Department of Neurology, Medical University of South Carolina, Charleston, SC, USA.

30. Neuroscience Department, University of Florence, Florence, Italy.

31. The Wales Epilepsy Unit, Department of Neurology, University Hospital of Wales, Cardiff, UK.

32. Neuroscience Institute, University of Cape Town, Cape Town, South Africa.

33. Florey Institute of Neuroscience and Mental Health, Austin Campus, Heidelberg, VIC, Australia.

34. University of Melbourne, Parkville, VIC, Australia.

35. Department of Neurology, Austin Health, Heidelberg, VIC, Australia.

36. Imaging Genetics Center, Mark and Mary Stevens Neuroimaging and Informatics Institute, Keck School of Medicine, University of Southern California, Marina del Rey, CA, USA.

37. Institute of Systems, Molecular and Integrative Biology, University of Liverpool, Liverpool, UK.

38. Department of Psychiatry University of Maryland School of Medicine, Baltimore, MD, USA.

39. Department of Clinical Neurophysiology, University Hospital Göttingen, Goettingen, Germany.

40. Department of Neurology and Epileptology, Hertie Institute for Clinical Brain Research, University of Tübingen, Tübingen, Germany.

41. University Medicine Göttingen, Clinical Neurophysiology, Göttingen, Germany.

42. Pediatric Neurology, Neurogenetics and Neurobiology Unit and Laboratories, Children’s Hospital A. Meyer‐University of Florence, Florence, Italy.

43. Functional and Epilepsy Neurosurgery Unit, Neurosurgery Department, Children’s Hospital A. Meyer-University of Florence, Florence, Italy.

44. Department of Neurology, The Royal Melbourne Hospital, Melbourne, VIC, Australia.

45. The Department of Medicine, The Royal Melbourne Hospital, The University of Melbourne, Parkville, VIC, Australia.

46. ‘Mario Serio’ Department of Clinical and Experimental Medica Sciences, University of Florence, Florence, Italy.

47. Psychiatric Genetics, QIMR Berghofer Medical Research Institute, Brisbane, QLD, Australia.

48. Department of Biomedical, Metabolic, and Neural Sciences, University of Modena and Reggio Emilia, Modena, Italy.

49. Neurology Unit, OCB Hospital, Azienda Ospedaliera-Universitaria Modena, Modena, Italy.

50. Department of Neurology, University of Campinas - UNICAMP, Campinas, SP, Brazil.

51. Cleveland Clinic Neurological Institute, Cleveland, USA.

52. School of Computing, Newcastle University, Newcastle upon Tyne, UK.

53. Division of Neuroscience, King’s College London, London, UK.

54. IRCCS Istituto ‘Giannina Gaslini’, Genova, Italy.

55. Department of Neurosciences, Rehabilitation, Ophthalmology, Genetics, Maternal and Child Health, University of Genova, Genova, Italy.

56. Department of Epileptology, University Hospital Bonn, Bonn, Germany.

57. Department of Neuroscience, Central Clinical School, Alfred Hospital, Monash University, Melbourne, VIC, Australia.

58. Departments of Medicine and Radiology, The Royal Melbourne Hospital, The University of Melbourne, Parkville, VIC, Australia.

59. School of Electrical and Computer Engineering, College of Engineering, University of Tehran, Tehran, Iran.

60. SA MRC Unit on Risk & Resilience in Mental Disorders, Dept of Psychiatry & Neuroscience Institute, University of Cape Town, Cape Town, South Africa.

61. Primary Care Department, Azienda Sanitaria Locale di Modena, Modena, Italy.

62. Institute of Experimental Epileptology and Cognition Research, University of Bonn, Bonn, Germany.

63. Support Center for Advanced Neuroimaging, University Institute of Diagnostic and Interventional Neuroradiology, Inselspital, Bern University Hospital, University of Bern, Bern, Switzerland.

64. Department of Medicine, Division of Neurology, Queen's University, Kingston, ON, Canada

1. Department of Radiology of Center of Image Diagnosis (CDIC), Hospital Clinic de Barcelona, Barcelona, Spain.

66. Centro Investigación Biomédica en Red de Salud Mental (CIBERSAM), Madrid, Spain.

67. Institute of Translational and Clinical Research, Newcastle University, Newcastle upon Tyne, UK.


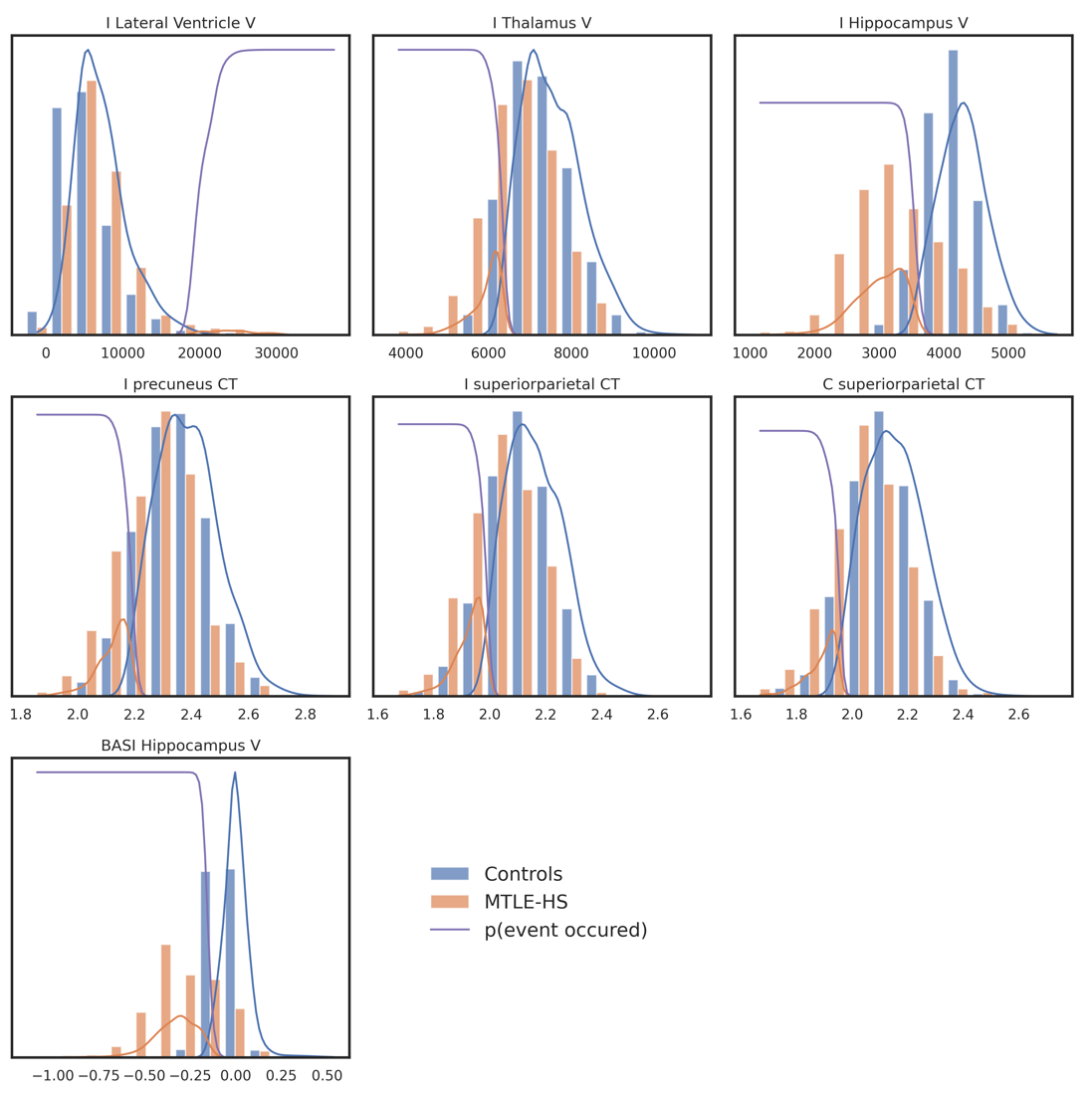


*Supplementary Figure S 1 Mixture models for the imaging biomarkers*: Histograms of the biomarker distribution for cases (orange) and controls (blue). The estimated density using KDE is indicated in solid lines of the same color. The resulting probability that the event has occurred (i.e., the biomarker value is considered abnormal) is indicated by a purple solid line.


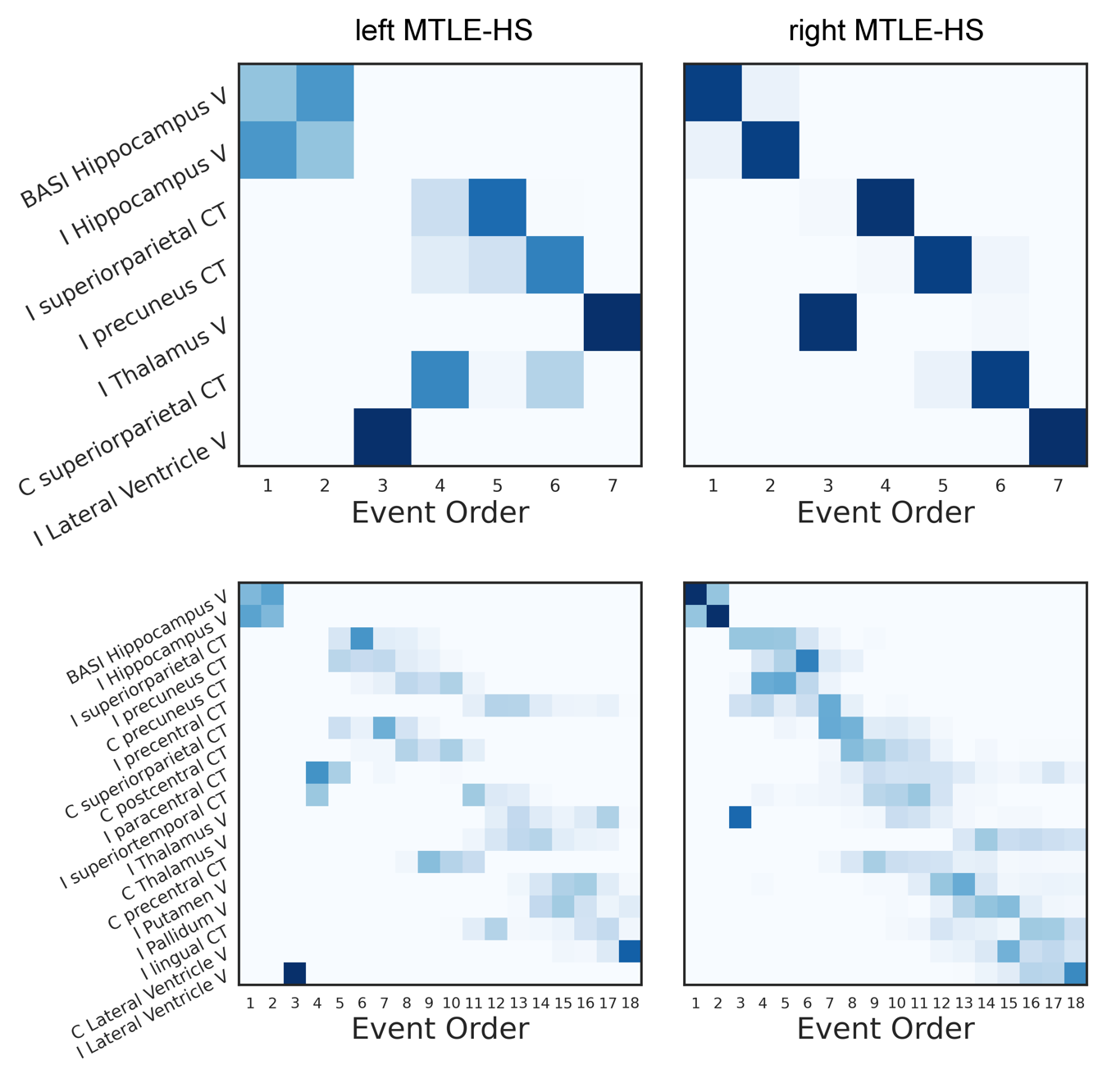


*Supplementary Figure S 2* *Positional variance diagram of left and right MTLE-HS cases*: The EBM was estimated using only left MTLE-HS cases (left column) or only right MTLE-HS cases (right column). The top row shows the results with the stringent inclusion criterion (|d| > 0.5) and the bottom row the relaxed inclusion criterion (|d|>0.4). To assist comparisons of the biomarker order, the y-axis ordering was based on the full EBM in Figure 3 for |d|>0.5 and Supplementary Figure S3 for |d|>0.4. The left MTLE-HS follows roughly the same ordering as the full EBM as indicated by most biomarkers following the diagonal (Spearman’s *ρ=0.39* for stringent and *ρ=0.63* for relaxed inclusion criteria, respectively). However, increase in ipsilateral lateral ventricle volume was placed very early in the sequence. The right MTLE-HS PVD shows a better alignment with the full EBM (Spearman’s *ρ=0.89* for stringent and *ρ=0.95* for relaxed inclusion criteria, respectively) than the left MTLE-HS model and indicates a preferred start with hippocampal volume asymmetry and, in addition, places reduced ipsilateral thalamic volume very early in the sequence. CT=cortical thickness, V=Volume, BASI=Brain Asymmetry Index, I=ipsilateral, C=contralateral.


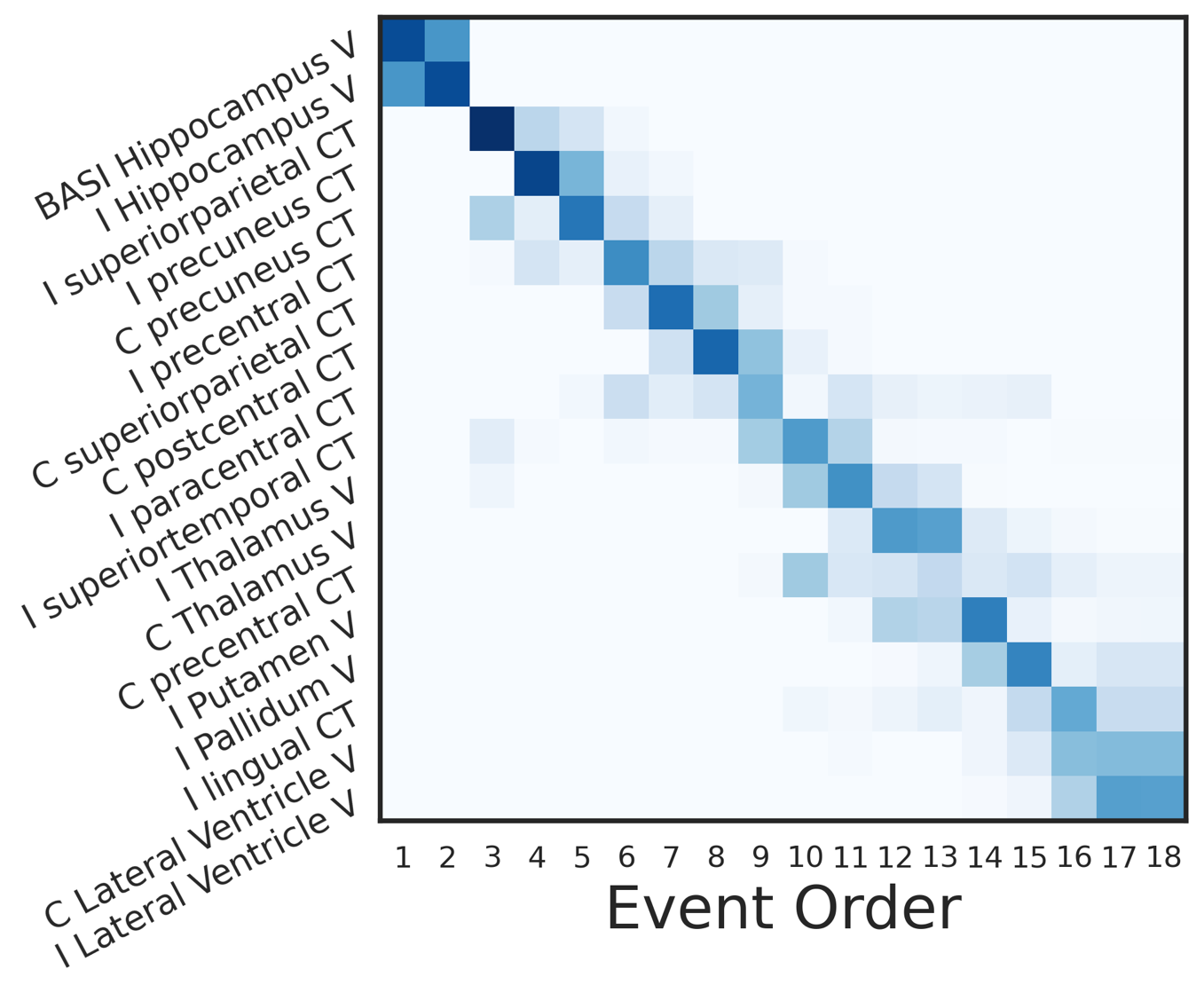


*Supplementary Figure S 3 Positional variance diagram for features above Cohen’s d of 0.4:* Positional variance diagram for the full dataset with 19 features passing the more lenient Cohens’ d cut-off (|*d*| ≥0.4). Fitting of the KDE mixture model for the biomarker ‘C caudalmiddlefrontal CT’ failed and the feature was therefore excluded from EBM modelling, leaving 18 biomarkers in the model. The ordering agrees with the original EBM: hippocampal features are followed by reduced cortical thickness, mainly in the parietal and frontal lobes. Next, there is a reduction in bilateral thalamic volumes and other subcortical structures. At the end of the sequence there is an increase in bilateral lateral ventricle volume. CT=cortical thickness, V=Volume, BASI=Brain Asymmetry Index, I=ipsilateral, C=contralateral.


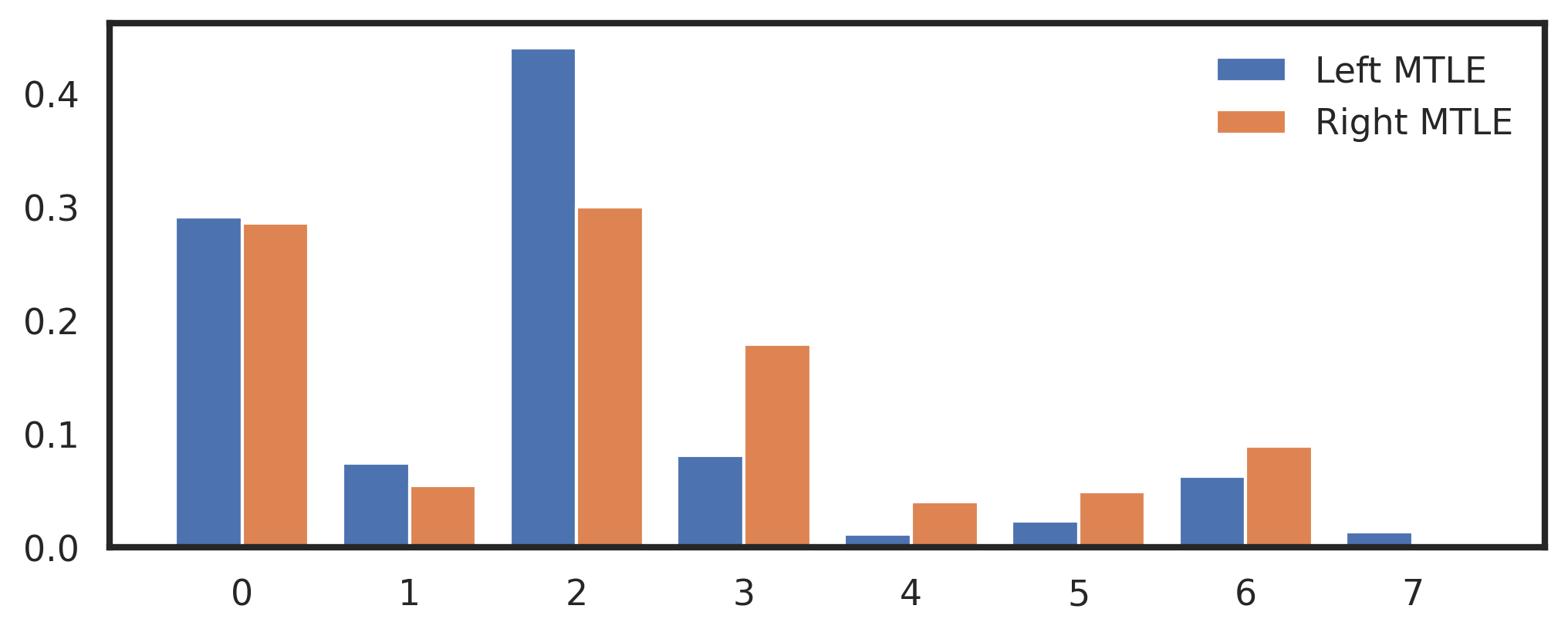


*Supplementary Figure S 4: Barplot of EBM stage distribution by diagnosis:* The x-axis shows the EBM stage and the y-axis the proportion of left MTLE-HS cases (blue) and right MTLE-HS cases (orange) assigned to these stages. A higher proportion of left MTLE-HS cases is assigned to stage 2 compared to right MTLE-HS cases. Conversely, right MTLE-HS cases were assigned more frequently to stages 3-7 compared to left MTLE-HS cases.


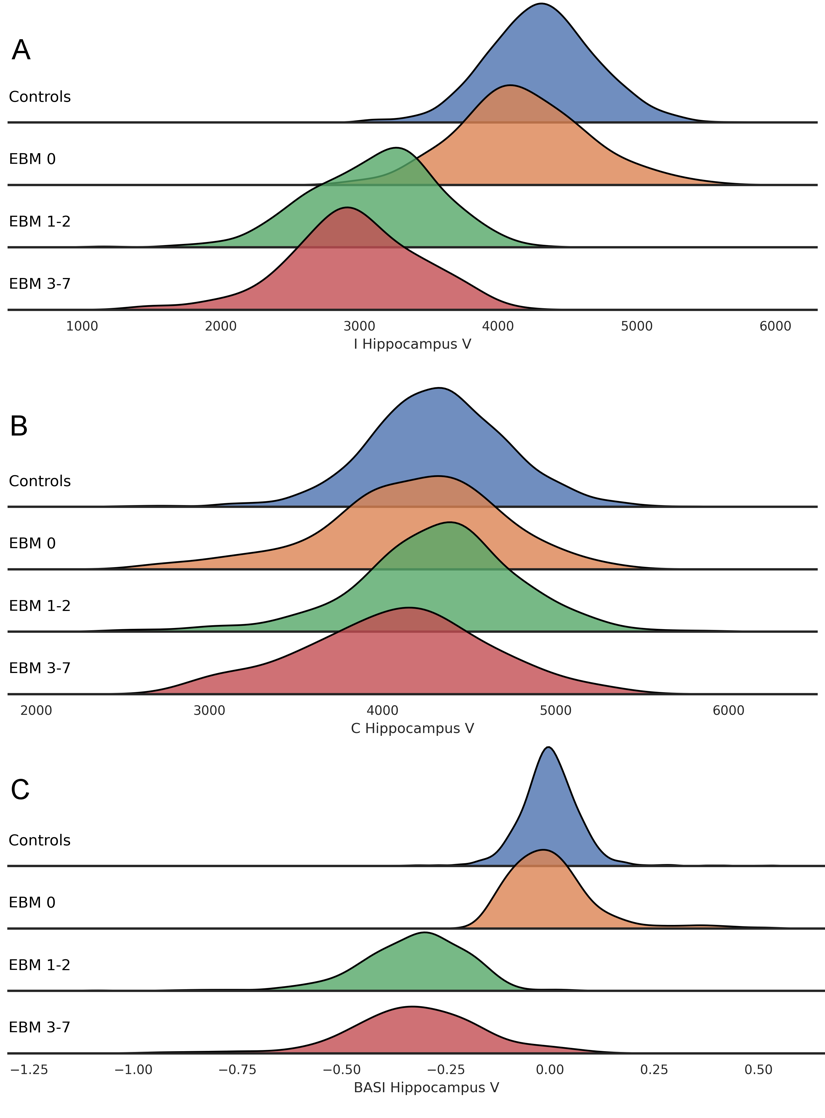


*Supplementary Figure S 5 Ridgeplots of hippocampal features in controls and MTLE-HS cases at different EBM stages:* Each panel shows a series of density plots for hippocampal features, i.e., ipsilateral hippocampal volume (**A**), contralateral hippocampal volume (**B**) and BAIS of hippocampal volume (**C**). In each panel subjects are grouped into Controls and (top ridge) and EBM stages for MTLE-HS cases. For MTLE cases at stage 0 (EBM 0), hippocampal asymmetry is the same as in controls and ipsilateral hippocampal volume is only slightly decreased compared to controls. MTLE cases at stages 1 and 2 (EBM 1-2) show increased hippocampal volume asymmetry, reduction in ipsilateral volume but no decrease in contralateral hippocampal volume.


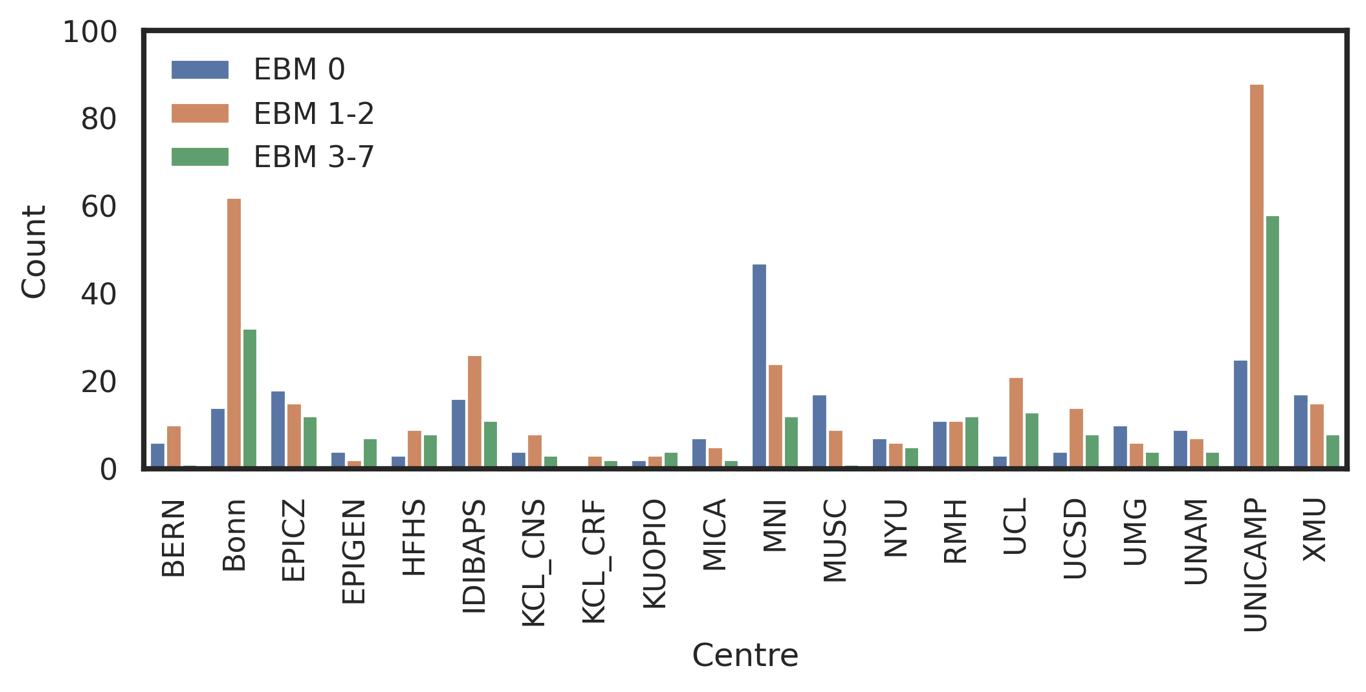


*Supplementary Figure S 6 Distribution of stages in MTLE-HS cases by contributing centre:* Distribution of MTLE-HS cases placed in stage 0, stages 1-2 and stages 3-7 from individual centres. Some centres have a high fraction of MTLE-HS cases with EBM stage 0 (blue bars) potentially indicating different protocols and sensitivities in establishing sclerosis of the hippocampus or the mesial temporal lobe.

*Supplementary Table S 1 List robust Cohen’s d for all features:* Features selected for the EBM are highlighted in bold and brown font. Features additionally selected for the sensitivity analysis are highlighted in blue font. BASI=Brain Asymmetry Index; I=ipsilateral; C=contralateral, CT=cortical thickness, SA=surface area, V=volume.

| Feature | Robust Cohen’s d |
| --- | --- |
| **BASI Hippocampus V** | **-2.6829179** |
| **I Hippocampus V** | **-1.7582463** |
| **I Thalamus V** | **-0.6664918** |
| **I precuneus CT** | **-0.5467175** |
| **I superiorparietal CT** | **-0.5380512** |
| **C superiorparietal CT** | **-0.5209357** |
| I superiortemporal CT | -0.4723382 |
| I lingual CT | -0.4631141 |
| I Pallidum V | -0.458555 |
| I Putamen V | -0.4504646 |
| C precentral CT | -0.4451158 |
| C precuneus CT | -0.4443393 |
| C Thalamus V | -0.4344409 |
| C postcentral CT | -0.4331437 |
| I precentral CT | -0.4222914 |
| I paracentral CT | -0.4218226 |
| C caudalmiddlefrontal CT | -0.4162054 |
| I superiorfrontal CT | -0.3899213 |
| I postcentral CT | -0.3874891 |
| BASI Amygdala V | -0.3866285 |
| I lateraloccipital CT | -0.3854351 |
| C supramarginal CT | -0.3842853 |
| C paracentral CT | -0.3835202 |
| C superiorfrontal CT | -0.3805965 |
| I supramarginal CT | -0.3803446 |
| C lateraloccipital CT | -0.3801412 |
| I caudalmiddlefrontal CT | -0.378605 |
| C Putamen V | -0.3742566 |
| I middletemporal SA | -0.3738439 |
| C Pallidum V | -0.3705729 |
| I inferiorparietal CT | -0.3645912 |
| I cuneus CT | -0.3626711 |
| C inferiorparietal CT | -0.3520396 |
| I parstriangularis CT | -0.3443501 |
| C cuneus CT | -0.3397424 |
| I fusiform CT | -0.3375276 |
| I inferiortemporal SA | -0.3368785 |
| I pericalcarine CT | -0.3275078 |
| C parsopercularis CT | -0.3188425 |
| C transversetemporal CT | -0.3119499 |
| C pericalcarine CT | -0.3117247 |
| BASI middletemporal SA | -0.3027246 |
| I middletemporal CT | -0.3017855 |
| I parahippocampal CT | -0.2930618 |
| BASI inferiorparietal SA | -0.2891688 |
| I parsopercularis CT | -0.2889303 |
| I Caudate V | -0.2880701 |
| I temporalpole CT | -0.27784 |
| I transversetemporal CT | -0.2759336 |
| I inferiorparietal SA | -0.2683861 |
| I temporalpole SA | -0.2677421 |
| I bankssts CT | -0.2596088 |
| C lingual CT | -0.2581602 |
| C rostralmiddlefrontal CT | -0.2566672 |
| C parstriangularis CT | -0.2522852 |
| I entorhinal CT | -0.2500147 |
| BASI inferiortemporal SA | -0.2483479 |
| BASI temporalpole CT | -0.2452617 |
| I precentral SA | -0.2407922 |
| BASI Thickness | -0.2351717 |
| I Amygdala V | -0.228986 |
| BASI superiortemporal SA | -0.2254611 |
| BASI parahippocampal CT | -0.2192267 |
| BASI Thalamus V | -0.2173022 |
| C parsorbitalis CT | -0.2121693 |
| I parstriangularis SA | -0.2120204 |
| I parahippocampal SA | -0.2068277 |
| BASI parsorbitalis SA | -0.206631 |
| BASI lingual CT | -0.2051527 |
| BASI rostralmiddlefrontal SA | -0.2017726 |
| I cuneus SA | -0.2004569 |
| BASI entorhinal CT | -0.1995361 |
| BASI frontalpole SA | -0.1971317 |
| BASI superiorparietal SA | -0.1962724 |
| I parsorbitalis SA | -0.1933529 |
| BASI superiortemporal CT | -0.1901711 |
| BASI fusiform CT | -0.189665 |
| I rostralmiddlefrontal SA | -0.1878315 |
| I SurfArea | -0.1862529 |
| C Caudate V | -0.1860718 |
| C posteriorcingulate CT | -0.1853445 |
| C bankssts CT | -0.1838757 |
| I rostralmiddlefrontal CT | -0.1803568 |
| C superiortemporal CT | -0.1774301 |
| I superiortemporal SA | -0.1753831 |
| I postcentral SA | -0.1727137 |
| I insula SA | -0.1698279 |
| I bankssts SA | -0.1677565 |
| BASI cuneus SA | -0.1673264 |
| BASI Putamen V | -0.1672498 |
| I inferiortemporal CT | -0.1627242 |
| C precentral SA | -0.1606092 |
| BASI inferiortemporal CT | -0.1584654 |
| C lateralorbitofrontal CT | -0.1566637 |
| C parsopercularis SA | -0.1562873 |
| BASI SurfArea | -0.1559051 |
| BASI parstriangularis SA | -0.1551918 |
| C parahippocampal SA | -0.1540064 |
| BASI bankssts SA | -0.1514161 |
| I lateralorbitofrontal SA | -0.1503787 |
| I superiorfrontal SA | -0.1458241 |
| C caudalanteriorcingulate CT | -0.1451751 |
| C Hippocampus V | -0.14319 |
| C middletemporal CT | -0.1426917 |
| BASI temporalpole SA | -0.1327397 |
| I Accumbens V | -0.1327248 |
| I parsorbitalis CT | -0.1292584 |
| BASI middletemporal CT | -0.1290904 |
| BASI superiorparietal CT | -0.1250625 |
| C caudalmiddlefrontal SA | -0.1235376 |
| I superiorparietal SA | -0.1226229 |
| C frontalpole CT | -0.1204295 |
| I posteriorcingulate CT | -0.1192416 |
| I lateralorbitofrontal CT | -0.1176395 |
| BASI posteriorcingulate SA | -0.116049 |
| BASI paracentral SA | -0.1130479 |
| I entorhinal SA | -0.1126223 |
| BASI pericalcarine SA | -0.1126034 |
| C medialorbitofrontal CT | -0.1072757 |
| C rostralanteriorcingulate SA | -0.1068092 |
| I caudalanteriorcingulate SA | -0.1067414 |
| C fusiform CT | -0.1062734 |
| BASI parahippocampal SA | -0.1049692 |
| BASI lateraloccipital CT | -0.1026907 |
| I lateraloccipital SA | -0.0948732 |
| C transversetemporal SA | -0.0938629 |
| C lateraloccipital SA | -0.093557 |
| C Accumbens V | -0.0913876 |
| C insula CT | -0.0903123 |
| C temporalpole CT | -0.0894061 |
| I posteriorcingulate SA | -0.0883885 |
| BASI fusiform SA | -0.0881418 |
| I fusiform SA | -0.0879827 |
| I caudalmiddlefrontal SA | -0.0860714 |
| I parsopercularis SA | -0.0859082 |
| C insula SA | -0.0857329 |
| BASI precentral SA | -0.0849061 |
| C fusiform SA | -0.0848152 |
| C paracentral SA | -0.0844464 |
| C isthmuscingulate SA | -0.0815205 |
| C postcentral SA | -0.080863 |
| I frontalpole CT | -0.0797915 |
| BASI Caudate V | -0.0771777 |
| BASI precuneus SA | -0.0754572 |
| C entorhinal CT | -0.0751199 |
| I medialorbitofrontal SA | -0.0742784 |
| I medialorbitofrontal CT | -0.0736646 |
| BASI entorhinal SA | -0.0723618 |
| C rostralmiddlefrontal SA | -0.0722609 |
| I isthmuscingulate CT | -0.0714677 |
| BASI medialorbitofrontal SA | -0.070861 |
| BASI superiorfrontal SA | -0.0703944 |
| C parahippocampal CT | -0.068767 |
| BASI insula SA | -0.0646701 |
| C posteriorcingulate SA | -0.0630943 |
| I pericalcarine SA | -0.060347 |
| C superiorfrontal SA | -0.0600856 |
| BASI precuneus CT | -0.0591003 |
| BASI pericalcarine CT | -0.0566343 |
| BASI lateralorbitofrontal SA | -0.0537998 |
| C caudalanteriorcingulate SA | -0.0529875 |
| C inferiortemporal SA | -0.0424029 |
| I paracentral SA | -0.0412793 |
| C superiortemporal SA | -0.0412381 |
| BASI postcentral SA | -0.0396793 |
| C lateralorbitofrontal SA | -0.0396383 |
| BASI lateraloccipital SA | -0.039085 |
| BASI paracentral CT | -0.0382261 |
| BASI bankssts CT | -0.035709 |
| I precuneus SA | -0.0351027 |
| C cuneus SA | -0.0336586 |
| C temporalpole SA | -0.0302501 |
| BASI caudalanteriorcingulate SA | -0.0275075 |
| BASI Pallidum V | -0.0233492 |
| BASI supramarginal SA | -0.0200667 |
| BASI caudalmiddlefrontal SA | -0.017667 |
| BASI superiorfrontal CT | -0.0157578 |
| I frontalpole SA | -0.015389 |
| C middletemporal SA | -0.0131659 |
| C entorhinal SA | -0.0116351 |
| C isthmuscingulate CT | -0.0115805 |
| BASI frontalpole CT | -0.0088867 |
| BASI Accumbens V | -0.0065703 |
| BASI parstriangularis CT | -0.0056471 |
| C inferiortemporal CT | -0.0009267 |
| I lingual SA | 0.00300391 |
| BASI parsopercularis CT | 0.0077404 |
| I insula CT | 0.0096573 |
| BASI postcentral CT | 0.00979354 |
| BASI transversetemporal CT | 0.00997855 |
| BASI posteriorcingulate CT | 0.01070268 |
| BASI inferiorparietal CT | 0.01107456 |
| BASI precentral CT | 0.01665731 |
| BASI rostralanteriorcingulate CT | 0.01928661 |
| C superiorparietal SA | 0.02132597 |
| C lingual SA | 0.02277294 |
| BASI isthmuscingulate CT | 0.02439135 |
| C SurfArea | 0.02487679 |
| I transversetemporal SA | 0.02490681 |
| C parsorbitalis SA | 0.02745711 |
| C bankssts SA | 0.02800504 |
| BASI medialorbitofrontal CT | 0.02888847 |
| BASI lateralorbitofrontal CT | 0.03268548 |
| C precuneus SA | 0.03765065 |
| C medialorbitofrontal SA | 0.03786681 |
| BASI supramarginal CT | 0.03942664 |
| BASI lingual SA | 0.0405 |
| BASI cuneus CT | 0.04124328 |
| C pericalcarine SA | 0.04423045 |
| BASI parsorbitalis CT | 0.04947811 |
| I caudalanteriorcingulate CT | 0.05240513 |
| C supramarginal SA | 0.07132833 |
| I supramarginal SA | 0.07164339 |
| I isthmuscingulate SA | 0.07213289 |
| C rostralanteriorcingulate CT | 0.07223438 |
| I rostralanteriorcingulate SA | 0.08276231 |
| I rostralanteriorcingulate CT | 0.08474284 |
| C parstriangularis SA | 0.08665371 |
| BASI caudalmiddlefrontal CT | 0.08741198 |
| C inferiorparietal SA | 0.09426966 |
| BASI isthmuscingulate SA | 0.09982338 |
| BASI rostralmiddlefrontal CT | 0.10202985 |
| BASI insula CT | 0.11399903 |
| BASI parsopercularis SA | 0.13987967 |
| BASI caudalanteriorcingulate CT | 0.15544052 |
| C frontalpole SA | 0.17046372 |
| BASI rostralanteriorcingulate SA | 0.17375967 |
| BASI Lateral Ventricle V | 0.18917073 |
| BASI transversetemporal SA | 0.20267358 |
| C Amygdala V | 0.2042172 |
| I Thickness | 0.20783915 |
| C Thickness | 0.21171169 |
| C Lateral Ventricle V | 0.44088061 |
| **I Lateral Ventricle V** | **0.5389778** |

*Supplementary Table S2: Association between availability of ASM data and demographic and clinical variables in the ENIGMA-Epilepsy cohort.*

|  | **Sex** | **Diagnosis** | **Age** | **Duration of illness** | **Age of onset** | **Site** |
| --- | --- | --- | --- | --- | --- | --- |
| **Test** | Chi-sq | Chi-sq | t-test | t-test | t-test | Chi-sq |
| **Statistic** | 1.02; df=1 | 0.67; df=1 | -2.54 | -4.96 | 3.49 | 426; df=19 |
| **Mean difference** |  |  | -2.08 | -5.26 | 3.02 |  |
| **p-value** | 0.31 | 0.41 | 0.011 | 8.88x10^-07^ | 0.0005 | 1.11x10^-78^ |

Columns correspond to the examined demographic and clinical variables. Diagnosis refers to left or right MTLE-HS. Rows: ‘Test’ indicates the applied test (Chi squared test or t-test); ‘Statistic’ provides the test statistic (and degrees of freedom (df); ‘Mean difference’ provides the difference of the variable between PWE with missing AMS response data and PWE with available response data; ‘p-value’ provides the resulting p-value.
